# Removal of Organic Dyes, Polymers and Surfactants Using Carbonaceous Materials Derived from Walnut Shells

**DOI:** 10.3390/ma17091987

**Published:** 2024-04-25

**Authors:** Małgorzata Wiśniewska, Teresa Urban, Karina Tokarska, Paulina Marciniak, Anna Giel, Piotr Nowicki

**Affiliations:** 1 Department of Radiochemistry and Environmental Chemistry, Institute of Chemical Sciences, Faculty of Chemistry, Maria Curie-Sklodowska University in Lublin, M. Curie-Sklodowska Sq. 3, 20-031 Lublin, Poland; teresa.urban@mail.umcs.pl (T.U.); karina.tok24@gmail.com (K.T.); 2 Department of Applied Chemistry, Faculty of Chemistry, Adam Mickiewicz University in Poznań, Uniwersytetu Poznańskiego 8, 61-614 Poznań, Poland; paumar14@st.amu.edu.pl (P.M.); anna.giel@amu.edu.pl (A.G.)

**Keywords:** walnut shells, physical activation, chemical activation, activated biocarbons, adsorption, methylene blue, Triton X-100, poly(acrylic acid), poly(ethylene glycol), liquid phase purification

## Abstract

A series of new granular carbonaceous adsorbents was prepared via single-stage physical and chemical activation of walnut shells. Their suitability for removing various types of organic pollutants (represented by dyes, surfactants and water-soluble polymers) from the liquid phase was assessed. The activation of the precursor was carried out with CO_2_ and H_3_PO_4_ using conventional heating. Activated biocarbons were characterized in terms of chemical composition, acidic–basic nature of the surface, textural and electrokinetic properties as well as thermal stability. Depending on the type of activating agent used during the activation procedure, the obtained biocarbons differed in terms of specific surface area (from 401 to 1361 m^2^/g) and the type of porous structure produced (microporosity contribution in the range of 45–75%). Adsorption tests proved that the effectiveness of removing organic pollutants from the liquid phase depended to a large extent on the type of prepared adsorbent as well as the chemical nature and the molecular size of the adsorbate used. The chemically activated sample showed greater removal efficiency in relation to all tested pollutants. Its maximum adsorption capacity for methylene blue, poly(acrylic acid), poly(ethylene glycol) and Triton X-100 reached the levels of 247.1, 680.9, 38.5 and 61.8 mg/g, respectively.

## 1. Introduction

Nowadays, activated carbons have a wide and constantly growing application, which is due to many reasons related to the availability of precursors, a wide range of production procedures, specific properties and utilization possibilities [1,2,3]. The raw material base for the production of these carbonaceous solids is very wide, often including precursors of plant origin, which constitute waste biomass that is difficult to store and manage [4,5,6]. The process of synthesis of activated carbons by physical or chemical activation includes many available and proven procedures using a whole range of activating agents [7,8,9]. Moreover, the properties of these material can also be changed to a relatively wide extent by using appropriate chemical modifications, both during the production and after the activation processes. By selecting the appropriate type of precursor, activation method, type of activating agent, thermal conditions and time of activation process, it is possible to obtain very desirable physicochemical properties of activated carbons, which include mainly the developed porous structure, acidic–basic properties of the surface and excellent sorption abilities towards specific substances, both from liquid and gaseous phases [10,11]. Carbon materials used as adsorbents to remove hazardous impurities from aqueous solutions or capture carbon dioxide from exhausted gases must demonstrate some specific properties [12,13]. The most important of them are a high adsorption capacity towards the substance being removed, a high adsorption selectivity in relation to the compound separated from the mixture of other components, the maintenance of the sorption capacity during multiple adsorption–desorption cycles (economic parameter determining the frequency of adsorbent exchange), fast adsorption kinetics influencing the single-cycle time (an equilibration time preferably up to 1 h), the possibility of an efficient regeneration to reduce the costs of the entire process (the optimal adsorbate–adsorbent interactions should not be too weak or too strong (namely in the range 10–40 kJ/mol)), resistance to contaminants present in the systems (it must be stable, i.e., in the oxidizing environment) and a low cost of the carbonaceous adsorbent [12].

The practical use of activated carbon includes its basic forms, namely, granulated, formed (including the impregnated one) and powdered. Granulated carbons composed of irregularly shaped granules and powdered ones (resulting from the granules grinding) are used primarily in the purification of liquids. The main directions of their application are the treatment of drinking water in waterworks (improving color, taste and smell, removal of pesticides and humic compounds, catalytic removal of residual chlorine and ozone), wastewater treatment (catalytic reduction of parameters such as chemical oxygen demand or absorbable organochlorinated compounds), the purification of utility and industrial water (e.g., in the production of beverages) and the removal of organic compounds in the process of groundwater and land remediation [14,15,16]. In addition, their use includes the decoloring of food products (sugar, glucose), clarifying of syrups (in confectionery) and purification and decolorization of alcohols [17]. In pharmacy, granulated and powdered carbons find application in processes of cleaning and dewatering paraffins, waxes and glycerin, deoiling condensates and degreasing and decolorizing solvents [18]. Formed carbons occur in the form of cylinders whose length is at least twice their diameter. Impregnated carbon is usually formed carbon; nevertheless, its surface is covered with an appropriate chemical compound (impregnation makes chemisorption occurrence possible, which increases the effectiveness of the catalytic reaction) [19]. A filter bed with formed/impregnated activated carbon has a lower air resistance than that of the same height with granulated carbon; that is why this type of material is used primarily for gas purification. Their most important applications include the reduction of volatile organic compounds, purification of post-reaction gases from various types of pollutants, deodorization of air (from odor-generating compounds such as hydrogen sulfide and ammonia), purification of natural gas from mercury and biogas from hydrogen sulfide, as well as the purification of exhaust gases (removal of heavy metals (such as mercury, cadmium and thallium), dioxins and furans and the adsorption of sulfur and nitrogen oxides) [20,21,22].

Despite the widespread use of many well-developed procedures for the use of activated carbons, intensive research is constantly being conducted on the synthesis, properties and application possibilities of new activated carbons. This is due to the emergence of new types of pollutants that enter sewage along with the introduction of new drugs, cosmetics, plant protection products or the implementation of modern technologies in industry [23]. The processes of these substances’ removal can be controlled effectively if the mechanisms governing them are thoroughly understood [24]. Therefore, the basic research in this field is extremely important, as it is the first and necessary stage of using new carbonaceous materials on a large scale.

Taking the above into account, in the presented studies, we try to assess the usefulness of common walnut shells as a precursor of granular carbon adsorbents and to characterize the physicochemical parameters and adsorption capacity of the materials thus obtained. The impact of two completely different variants of thermochemical conversion of biomass on the chemical composition, thermal stability, as well as textural, electrokinetic and acidic–basic properties of the adsorbent was investigated. This approach aimed to select an adsorbent with greater application potential and to indicate the activation procedure, whose further optimization should bring the most benefits from a practical point of view. Therefore, the resulting activated biocarbons were used as potential adsorbents of various types of organic compounds, represented by methylene blue (MB, a synthetic cationic dye), poly(ethylene glycol) (PEG, a nonionic polymer), poly(acrylic acid) (PAA, an anionic polymer) and Triton ^TM^ X-100 (TX, a nonionic surfactant). Due to the wide use of the selected organic substances (textile, fur, tanning, paper and wood industries, pharmaceuticals, cosmetics and food production, paint and varnish technologies), their presence in industrial wastewater can be considerable [25]. They can enter surface water (and then the soil) and can cause toxic, mutagenic and/or carcinogenic effects in living organisms. Industrial wastewater has a very complex composition; that is why the surface behavior of dye, polymers and surfactant should be examined [26]. What is more, the possibility of removing water-soluble polymers using carbonaceous materials is extremely rarely investigated or limited to only one type of contamination.

## 2. Materials and Methods

### 2.1. Preparation of Carbonaceous Adsorbents

**Figure 1:**
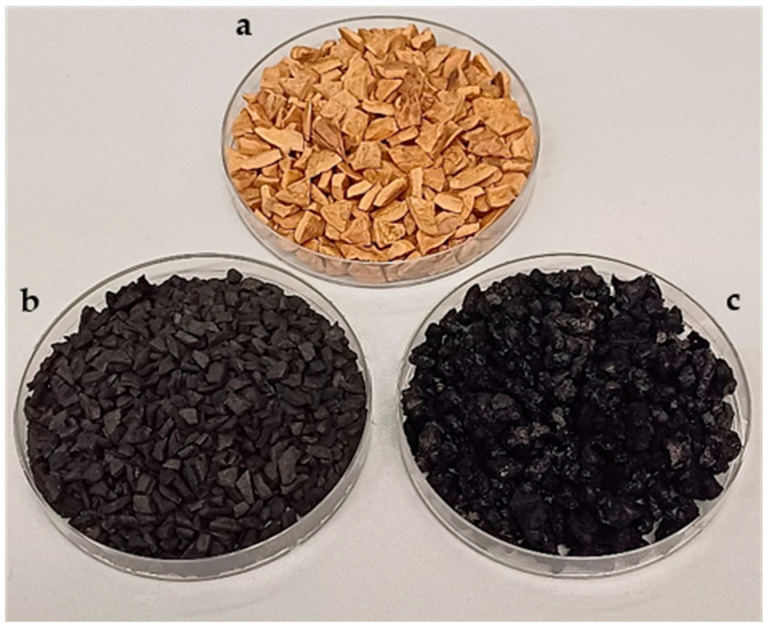
Starting walnut shells (**a**) and activated biocarbons obtained by direct (**b**) and chemical activation (**c**).

Walnut shells (*Juglans regia* L.), collected in the eastern part of the Wielkopolska region (Poland), were used as a precursor to obtain carbonaceous adsorbents. The starting material (WS) was crushed in a laboratory mill equipped with a cutting–grinding head (IKA Poland, Sp. z o. o., Warsaw, Poland) and sieved to a uniform grain size of 2–4 mm (Figure 1a). The fragmented shells were then subjected to two variants of the activation procedure: direct physical activation with CO_2_ (DPA, also called one-step physical or thermal activation) and chemical activation with H_3_PO_4_ (CA). Both activation variants were realized in a quartz tubular reactor heated by a single-zone laboratory resistance furnace (Czylok, Jastrzębie-Zdrój, Poland) using a conventional heating method.

Direct activation procedure: 20 g of the crushed walnut nutshells was placed in a nickel boat (length 150 mm and height/width 30 mm) and subjected to thermochemical conversion under a technical carbon dioxide atmosphere (CO_2_ 2.8 at a flow of 15 L/h; Linde Gaz Polska, Kościan, Poland). The precursor was heated (5 °C/min) from room temperature to a final activation temperature of 850 °C (to prevent an uncontrolled burn-off of biomass), annealed under these conditions for 30 min (in order to ensure the appropriate level of porous structure development) and then cooled down in a technical nitrogen atmosphere (N_2_ 4.0, at a flow of 10 L/h; Linde Gaz Polska, Kościan, Poland). The obtained activated biocarbon was designated as WSPDA (Figure 1b). The yield of the direct activation product was 22.5 wt.%.

Chemical activation procedure: 25 g of the precursor was impregnated with an 85% solution of orthophosphoric acid (Avantor Performance Materials, Gliwice, Poland) under the following conditions: walnut shells-activating agent’s weight ratio, 1:2; impregnation time, 24 h; temperature, 22 ± 1 °C; occasional mechanical mixing). The impregnated sample was then dried at a temperature of 110 °C, placed into a nickel boat and finally subjected to thermochemical treatment under a technical nitrogen atmosphere (flow rate, 20 L/h). The heating procedure consisted of the following stages:

(1) Heating the impregnated material to a temperature of 200 °C (heating rate of 5 °C/min);
(2) Annealing the material at 200 °C for a period of 30 min;
(3) Further heating the material to a final activation temperature of 500 °C (heating rate of 5 °C/min);
(4) Annealing the carbonaceous material at 500 °C for a period of 30 min;
(5) Cooling the activation product to room temperature.

Finally, the sample was washed with 10 L of boiling demineralized water and dried at 110 °C for 12 h. The obtained activated biocarbon was denoted as WSCA (Figure 1c). The yield of the chemical activation product was 49.7 wt.%.

### 2.2. Physicochemical Characterization of the Precursor and Carbonaceous Adsorbents

#### 2.2.1. Proximate and Elemental Analysis

The elemental composition of the starting walnut shells and both activated biocarbons was determined using a CHNS Vario EL III elemental analyzer (Elementar Analysensysteme GmbH, Langenselbold, Germany). The materials were also analyzed by X-ray fluorescence (ED-XRF Epsilon 5, PANalytical B.V., Almelo, The Netherlands). The ash contribution in the structure of the obtained materials was established using the Phoenix^TM^ microwave furnace (CEM Corporation, Matthews, IL, USA). The detailed procedure for these determinations was presented in a previous work [27]. The moisture and volatile matter contribution in the precursor structure was determined according to the PN-ISO 579:2002 [28] and PN-G-04516:1998 Standards [29], respectively.

#### 2.2.2. Textural Studies

Textural parameters of both activation products were determined on the Autosorb iQ sorptometer, manufactured by Quantachrome Instruments (Boynton Beach, FL, USA). Before measuring the N_2_ sorption isotherms, the activated biocarbon samples were outgassed under vacuum at 250 °C. The total surface area (S_t_) of activated biocarbons was calculated by the BET method (p/p_0_ range of 0.05–0.30). The total pore volume (V_t_) was calculated by converting the quantity of liquid nitrogen adsorbed at a p/p_0_ of 0.99. The mean pore diameter (D) was calculated from the relationship D = 4V_t_/S_t_. The micropore volume (V_micr_) and micropore area (S_micr_) were calculated using the t-plot method. The pore size distribution (PSD) was determined based on the BJH model, using the adsorption branch of the isotherm.

#### 2.2.3. Acid–Base Properties of the Surface

The content of acidic and basic functional groups formed during the activation process on the adsorbent surface was determined using the Boehm titration method, described in detail in our previous work [27]. Volumetric standards of 0.1 mol/dm^3^ NaOH and 0.1 mol/dm^3^ HCl (both provided by Avantor Performance Materials, Gliwice, Poland) were used as the titrants.

#### 2.2.4. Thermogravimetric Studies

A thermogravimetric analysis of the starting walnut shells and the resulting activated biocarbons was performed on a SETSYS 12 (Setaram, Caluire, France). The samples with a mass of ~10 mg and a particle size below 0.1 mm were heated from room temperature to 1000 °C (at the rate 10 °C/min), in a helium atmosphere.

#### 2.2.5. Surface Charge Density Determination

The surface charge density (σ_0_) of both activated biocarbons as well as their points of zero charge (pzc) were determined using the potentiometric titration method. For this purpose, 50 mL of suspensions containing 0.05 g of WSDPA or 0.015 g of WSCA activated biocarbon as well as an appropriate adsorbate with a concentration of 200 ppm were used. These measurements were performed at 25 °C in the pH range 3–11. Changes in the pH of the suspension after adding each portion of the titrant (0.1 M NaOH, Avantor Performance Materials, Gliwice, Poland) were monitored using a pHM 240 pH meter (Radiometer, Warsaw, Poland) equipped with glass and calomel electrodes (Beckman Instruments, Brea, CA, USA). Changes in the surface charge density value as a function of solution pH were calculated using “Titr_v3” application, described in detail in a previous paper [30].

#### 2.2.6. Electrophoretic Mobility Measurements

The electrophoretic mobility of activated biocarbon particles, enabling the determination of the zeta potential (ζ) and isoelectric point (iep), was performed using a Zetasizer Nano ZS, provided by Malvern Instruments (Malvern, United Kingdom). The zeta potential was calculated using Henry’s equation [31]. For this purpose, 200 mL of suspensions containing the tested polymers or surfactant with a concentration of 200 ppm and 0.03 g of the activated biocarbon were prepared. Each dispersed system was subjected to the action of ultrasound (XL 2020 ultrasonic head, Misonix, Farmingdale, NY, USA) and divided into 8 equal parts. The measurements were performed at 25 °C in the pH range 3–10. The appropriate pH value of the suspension was set using a 0.1 M HCl or NaOH solution (Avantor Performance Materials, Gliwice, Poland) and the Φ360 pH meter (Beckman, Brea, CA, USA). In this way, the zeta potential dependencies as a function of the solution pH were obtained.

### 2.3. Adsorption Studies

Four types of organic compounds of different chemical nature were used in order to characterize the sorption capacity of the walnut shells-based activated biocarbons. These were:

(1) A synthetic cationic dye with an aromatic character: methylene blue—MB (Avantor Performance Materials, Gliwice, Poland);
(2) Two water-soluble polymers of different ionic character: an anionic poly(acrylic acid)—PAA (Fluka, Saint Louis, MO, USA) and nonionic poly(ethylene glycol)—PEG (Sigma-Aldrich, Saint Louis, MO, USA), both with an average molecular weight equal to 2 kDa. PAA belongs to the group of weak polyelectrolytes and its carboxyl functional groups undergo a dissociation with the increasing solution pH. The pK_a_ value for PAA chains occurs at a pH of about 4.5 [32], at which the number of dissociated -COO^−^ and undissociated -COOH groups is the same (the dissociation degree is 0.5). At pH 3, the dissociation degree of PAA functional groups is minimal (about 0.03), so that the conformation of macromolecules is coiled [33]. In turn, starting from pH 6 towards higher pH values, the polyelectrolyte dissociation reaches a value close to 1 and polymeric chains assume a considerably developed conformation in the solution. In the case of nonionic PEG, whose chains contain ether oxygen atoms having free electron pairs and terminal hydroxyl groups, its conformation in solution is unchanged over the wide pH range.
(3) A nonionic surfactant, Triton ^TM^ X-100 (t-octylphenoxypolyethoxyethanol)—TX (Sigma-Aldrich, Saint Louis, MO, USA) with a molar mass in the range 602.80–646.85 g/mol. In its molecule, two parts can be distinguished: the hydrophobic substituent containing the phenolic ring and the hydrophilic polyoxyethylene fragment [34].

In the case of MB removal, the adsorption studies were performed according to the procedure described in detail in [35]. The influence of the initial concentration of methylene blue on the efficiency of its removal from aqueous and alcoholic solutions was checked. The initial and equilibrium dye concentrations were determined using a Cary 100 Bio UV–visible spectrophotometer (Agilent, Santa Clara, CA, USA) at the wavelength of 664 nm (in the case of an aqueous solution) and 656 nm (in the case of an alcoholic solution), using the previously prepared calibration curves. Distilled water or 96% ethanol (Avantor Performance Materials, Gliwice, Poland) was applied as a reference sample. All adsorption experiments were performed twice with a reproducibility of ±5%. The mean values of the results were used for the data evaluation.

The two most popular adsorption isotherm models (Langmuir and Freundlich, expressed by Equations (1) and (2)) were applied to analyze the equilibrium data:

(1) $$q=\frac{q_{max}K_{L}c}{1+K_{L}c}$$

(2) $$q=K_{F}c^{1/n}$$

with q_ads_—the equilibrium MB adsorbed amount [mg/g], q_max_—the maximum capacity of the adsorption monolayer [mg/g], K_L_—Langmuir constant [dm^3^/mg], c—the equilibrium dye concentration [mg/dm^3^], K_F_—Freundlich constant [mg/g (mg/dm^3^)^1/n^], n—Freundlich parameter determining the adsorption strength.

The kinetics of MB adsorption from aqueous and alcoholic solutions was also investigated. The procedure was similar to that used for the equilibrium tests. The suspension was stirred magnetically (200 rpm) for 6 h, at a temperature of 25 ± 1 °C. For a period of 2 h, samples were collected every 15 min and then every 30 min. The obtained results were fitted using the pseudo-1st and the pseudo-2nd-order kinetic models, expressed by Equations (3) and (4):

(3) $$\frac{{dq}_{t}}{dt}=k_{1}(q_{eq}-q_{t})$$

(4) $$\frac{{dq}_{t}}{dt}=k_{2}{\left(q_{eq}-q_{t}\right)}^{2}$$

with q_eq_—the amount of methylene blue adsorbed in the equilibrium state [mg/g], q_t_—the amount of dye adsorbed after time “t” [mg/g], k_1_—the pseudo-1st-order rate constant [1/min], k_2_—the rate constant of the pseudo-2nd-order adsorption [g/(mg·min)].

All adsorption experiments were performed twice with a reproducibility of ±5%. The mean values of the results were used for the kinetic data evaluation. A linear and a non-linear method for the calculation of kinetic parameters in adsorption studies were applied using the Microsoft Excel 2013 Solver software 14.0.7268.5000 add-in. Moreover, the analysis of Marquardt’s percent standard deviation (MPSD) as well as the determination coefficient (R^2^) values were utilized to find the best fitting model.

Adsorption tests for both polymers and surfactant were carried out at a temperature of 25 ± 1 °C in the pH range 3–11 (±0.1). The adsorbed amounts were determined by applying the static method (based on changes in the adsorbate concentration before and after the adsorption process). The initial concentration of all adsorbates was 200 ppm, the mass of activated biocarbons was 0.05 g, and the volume of the suspension was 10 mL. In the case of PEG, due to its complete adsorption on the WSCA surface under such conditions, the initial concentration range was increased to 1000 ppm. In this way, the maximal sorption capacity of the material obtained by chemical activation could be determined. Such a range of poly(ethylene glycol) concentration corresponds with its content in real industrial wastewaters. The methodology with hyamine 1622 proposed by Crummett and Hummel [36] was used for the determination of the poly(acrylic acid) concentration in the solution. The absorbance coming from the white-colored PAA-hyamine complex was measured at the wavelength of 500 nm after 15 min of the hyamine addition, using the UV–vis spectrophotometer Cary 100 (Varian, Palo Alto, CA, USA). In the case of poly(ethylene glycol) the complexation reaction of PEG with tannic acid elaborated by Nuysink and Koopal [37] was applied. After 15 min, the resulting turbidity was measured spectrophotometrically at a wavelength of 600 nm. In turn, the Triton ^TM^ X-100 concentration was determined directly, because this surfactant solution gives maximal absorbance without any indicators at a wavelength of 270 nm [38,39].

## 3. Results and Discussion

### 3.1. Proximate and Elemental Analysis of the Starting Walnut Shells and Products of Their Thermochemical Conversion

**Figure 2:**
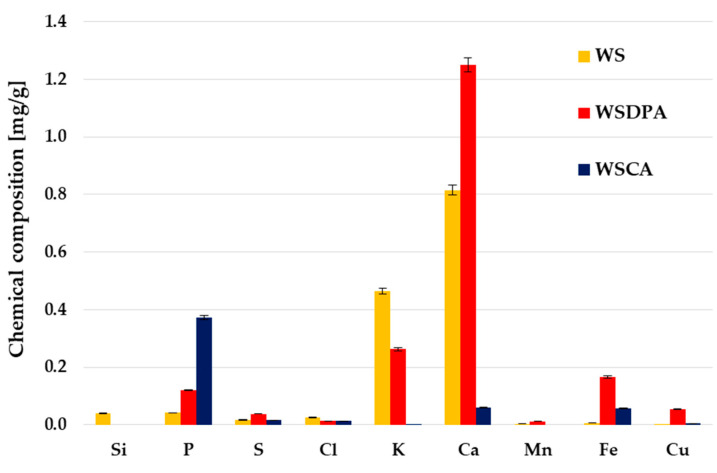
XRF analysis of the starting walnut shells and activated biocarbons obtained by direct and chemical activation.

The analysis of the data collected in Table 1 reveals that the starting walnut shells were characterized by a very low ash and moisture content (less than 1% by mass) and at the same time, a high contribution of elemental carbon in the structure, which makes them a suitable material for the production of carbonaceous adsorbents. A minor drawback was a high content of volatile matter (VM), which may have resulted in a lower yield of the physical activation process, which usually takes place at temperatures above 800 °C. The XRF analysis (Figure 2) showed that the precursor used for research also contained small amounts of Si, P, Cl, K, Ca, Mn, Fe and Cu, which is a quite typical trend for plant biomass.

Both activation variants led to intensive changes in the elemental composition compared to the precursor. As a result of the thermochemical treatment, especially in the presence of carbon dioxide (WSDPA sample), the share of elemental carbon increased. In turn, the content of other elements (in particular oxygen) was clearly reduced. The lower intensity of these changes observed in the case of the material activated with the orthophosphoric acid (WSCA sample) most probably resulted from the milder temperature regime (activation temperature lower by 350 °C). This was evidenced by the higher yield of the chemical activation product.

The activation procedure also caused changes in the content of inorganic admixtures. A high processing temperature and the use of CO_2_ as an activating agent led to an increase in the content of P, S, Ca, Fe and Cu and a simultaneous decrease in the content of Si and K, which was most likely a consequence of the distillation of the less thermally stable fragments of the carbon structure. In the case of a chemical activation of walnut shells, different relationships were observed. The phosphorus content in the WSCA sample was approximately four times higher than in the precursor, which was a consequence of the use of H_3_PO_4_ as an activating agent and its incorporation into the carbonaceous structure in the form of phosphate groups [40]. As a result of thermal treatment in the presence of an acidic activator, the content of metallic components (especially calcium and potassium) was reduced (due to their dissolution).

### 3.2. Textural and Surface Properties of Activated Biocarbons Derived from Walnut Shells

**Figure 3:**
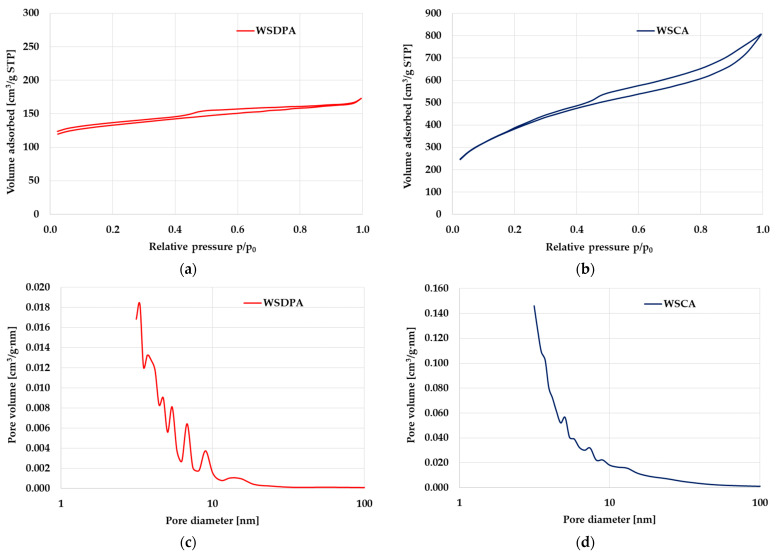
Low-temperature N_2_ adsorption/desorption isotherms (**a**,**b**) and pore size distribution (**c**,**d**) for WSDPA and WSCA samples.

The textural parameters and chemical nature of the surface are the most important physicochemical properties of activated biocarbons, especially from the adsorption point of view. As follows from the results shown in Table 2 and Figure 3, the textural parameters of the carbonaceous adsorbents prepared depended on the type of activating agent used for their production. The product of the chemical activation with H_3_PO_4_ had a well-developed surface area (1361 m^2^/g) and total pore volume (1.25 cm^3^/g) with a predominance of mesopores. The analogous material obtained via a one-stage physical activation with CO_2_ had less favorable textural parameters (S_t_ = 401 m^2^/g, V_t_ = 0.27 cm^3^/g), despite the much higher processing temperature. It should also be noted that the WSDPA sample was characterized by a clear predominance of micropores in its structure. This suggests that carbon dioxide is a much less reactive activating agent towards to the precursor used. It would probably be more advantageous to use walnut shells with a smaller grain size (or alternatively to use a higher temperature or longer activation time), but this would also involve a reduction in the yield of the activation product as well as a further increase in the mineral matter contribution in its structure [41]. It is worth emphasizing that the obtained activated biocarbons (especially the product of chemical activation) were characterized by quite favorable textural parameters compared to materials prepared using walnut shells with a much smaller initial grain size and more drastic activation conditions (Table 3).

The significant micropores contribution in the porous structure of the physically activated biocarbon was confirmed by the shape of the low-temperature N_2_ sorption isotherm shown in Figure 3a. Its course resembled the connection of type I and IV isotherms (according to the IUPAC classification) [48,49], which is characteristic of microporous and mesoporous adsorbents with a pore size close to the micropores’ range. The narrow hysteresis loop (H4 type) observed in its course indicated the presence of mesopores in the structure of the WSDPA sample. This type of hysteresis loop is most often attributed to materials containing narrow slit-like pores or internal voids of irregular shape and broad size distribution. In the case of the chemically activated biocarbon, N_2_ sorption isotherm shape was very similar to type IV (Figure 3b). The wide hysteresis loop visible in its course (in the p/p_0_ range of 0.45–1.00) confirmed the predominance of mesopores in the porous structure of the WSCA sample. The presence of small mesopores (with diameter in the range of 2–20 nm) in the structure of both activated biocarbons was confirmed by the PSD curves shown in Figure 3c,d.

**Figure 4:**
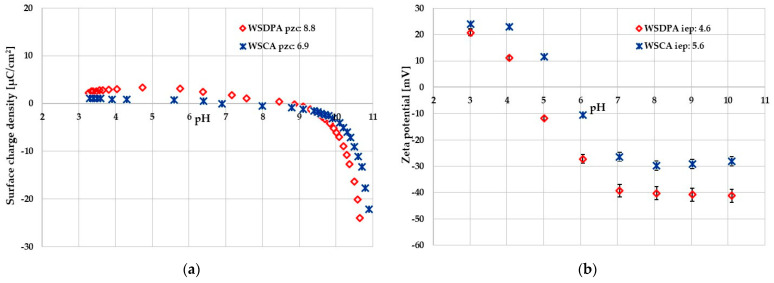
Surface charge density (**a**) and zeta potential (**b**) of activated biocarbons particles in an aqueous suspension.

The data shown in Table 2 and Figure 4 clearly indicate that carbonaceous materials obtained by chemical and direct activation of walnut shells have a completely different acidic–basic nature of their surface, which may determine their suitability for adsorption purposes. The sample activated with carbon dioxide at temperature of 850 °C contained almost exclusively basic functional groups on its surface. On the contrary, in the case of the WSCA sample prepared by chemical activation of walnut shells at 500 °C, a clear predominance of acidic functional groups (0.705 mmol/g) over basic ones (0.099 mmol/g) was observed. This indicates notable differences in the mechanism of interaction of both activating agents with biomass.

Figure 4 presents the pH dependence of the surface charge density (case (a)) and the zeta potential (case (b)) of both activated biocarbons particles dispersed in an aqueous medium. The points of intersection of the curves with the *x* axis, which correspond to surface charge density (σ_0_) and zeta potential (ζ) values equal to zero, allow for determination of the points of zero charge (pzc) and isoelectric points (iep) of carbonaceous materials, respectively. These points corresponds with the specific pH values, namely pH_pzc_ and pH_iep_, which are characteristic of each adsorbent–adsorbate system [50]. It was shown that the pH_pzc_ values for the WSDPA and WSCA samples were 8.8 and 6.9, respectively (Figure 4a), which confirmed the acidic nature of the surface of chemically activated material and the alkaline nature of the sample prepared via a direct activation of the walnut shells.

In the pH range below pH_pzc_, the surface of activated biocarbon is positively charged, and the adsorption of negatively charged adsorbates is electrostatically favored. In turn, at pH values above pH_pzc_, the solid surface charge has a negative sign, and the electrostatic attraction with positively charged adsorbates is promoted. The isoelectric points of the tested carbonaceous adsorbents were located at lower pH values and were equal to 4.6 for the WSDPA sample and 5.6 for the WSCA one (Figure 4b). Such a difference between the values of the pH_pzc_ and pH_iep_ points is observed in many systems and can be the result of a mutual overlapping of electrical double layers (EDLs) formed on the opposite walls of adsorbent pores, which affects the ionic composition of the diffusion EDL parts [51].

### 3.3. Thermal Stability of Activated Biocarbons Derived from Walnut Shells

**Figure 5:**
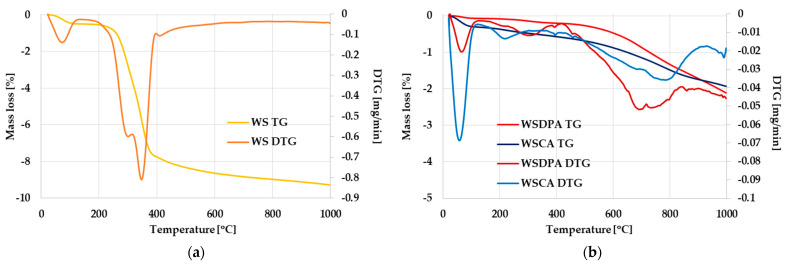
TG and DTG curves of walnut shells (**a**) and activated biocarbons obtained via (**b**).

The thermal properties of the tested materials were characterized using thermogravimetric measurements conducted in an inert gas (helium) atmosphere. The thermograms presented in Figure 5 clearly indicate that both activated biocarbons show a much higher thermal stability than the starting walnut shells. However, it should be noted that the WSDPA sample (obtained as a result of a direct activation of the precursor) was more resistant to high temperature than the WSCA biocarbon (activated with H_3_PO_4_), which was undoubtedly related to the thermal conditions of the activation procedure.

By comparing the course of the TG and DTG curves presented in Figure 5b, several similarities between individual materials can be observed. The thermal decomposition of both activated biocarbons takes place in three main stages. The first observed minimum (at ~60 °C) is most likely related to the evaporation of physically adsorbed water. The intensity of that peak is much higher in the case of the WSCA sample, which may indicate a greater hygroscopicity of its structure. The second DTG minimum, noticeable at 230 °C for WSCA and at 310 °C for the WSDPA sample, can be attributed to the decomposition of the least thermally stable oxygen functional groups (e.g., carboxyl or lactone) present in the structure of the prepared biocarbons. The last, very broad peak observed between 500 and 900 °C can be attributed to a further defragmentation of surface functional groups (e.g., carbonyl, phenolic, ether or carbonate ones) and the gradual gasification of the least thermally stable fragments of the carbonaceous structure.

### 3.4. Adsorption of a Cationic Synthetic Dye from an Aqueous and Alcoholic Solution

**Figure 6:**
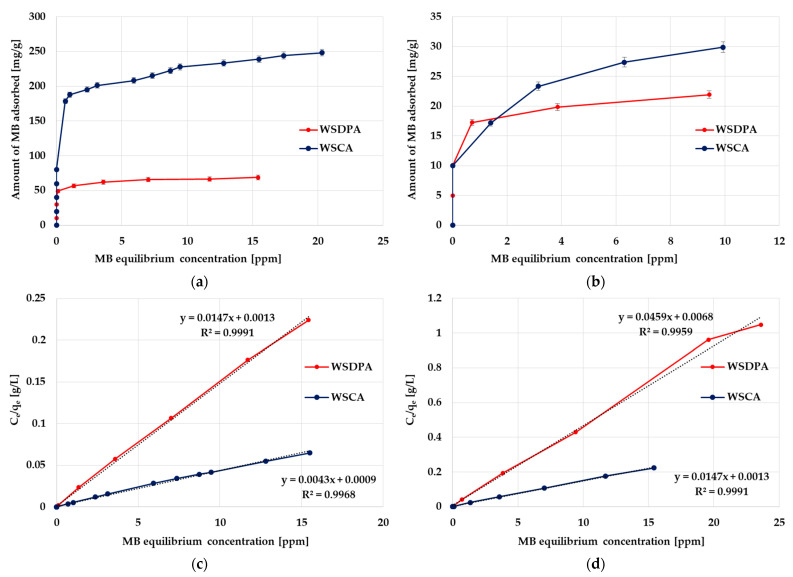
The equilibrium isotherms of methylene blue adsorption from aqueous solution (**a**) and alcoholic solution (**b**) as well as the corresponding Langmuir isotherm plots (**c**,**d**).

The first variant for assessing the sorption abilities of the obtained activated biocarbons was the adsorption of methylene blue (a synthetic cationic thiazine dye) from an aqueous and alcoholic solution. The results of adsorption tests, summarized in Figure 6 and Table 4, indicated unequivocally that the products of direct and chemical activation of the walnut shells showed very diverse efficiency values of this organic dye removal. Both of the tested carbonaceous materials were able to adsorb a greater amount of methylene blue from an aqueous medium. However, a much more effective adsorbent (especially in an aqueous environment) was the H_3_PO_4_-activated sample, which is most likely due to its highly developed porous structure. It turned out that in the case of adsorption from the ethanol/MB/activated biocarbon system, the influence of the textural parameters of the solid was much less important, as evidenced by the relatively similar sorption capacities of WSDPA and WSCA carbons (21.9 and 29.7 mg/g, respectively). This is probably a consequence of differences in the solubility of methylene blue in these two solvents and in the value of the surface tension of these systems.

The analysis of the data presented in Table 4 may imply that the Langmuir isotherm fitted the experimental data more accurately than the Freundlich one. This was suggested by the higher and close-to-unity values of the determination coefficient R^2^ for this model, especially in the case of MB removal from an alcoholic solution. It was also indicated by comparable values of the experimental (q_exp_) and calculated (q_max_) sorption capacities. However, the high R^2^ values for the Freundlich model observed in case of the MB adsorption from an aqueous medium (0.979–0.989) suggested that a slightly more complicated mechanism of adsorption (including multilayer adsorption as a result of interactions between adsorbate molecules) could not be ruled out.

**Figure 7:**
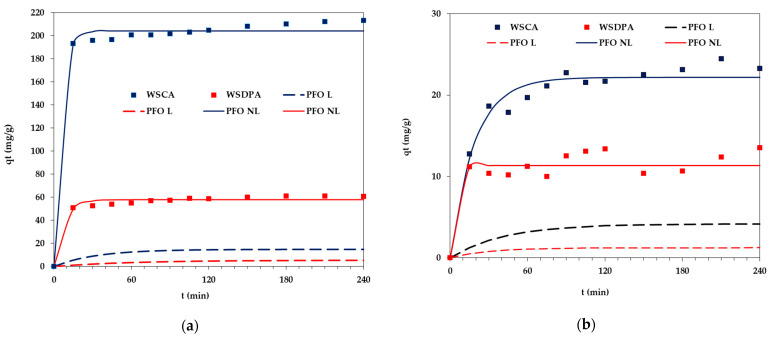
Effect of phase contact time on methylene blue adsorption from an aqueous solution (**a**) and an alcoholic solution (**b**) (L—linear fitting, NL—non-linear fitting).

The effect of contact time on the removal of methylene blue from an aqueous and alcoholic solution was also investigated. According to the data presented in Figure 7, the kinetics of methylene adsorption consisted of two stages: (1) the initial rapid phase, in which the instantaneous adsorption of methylene blue or covering of the external adsorbent surface with adsorbate molecules took place (contributing to the equilibrium dye uptake) and (2) a slow second stage during which the gradual dye adsorption leading to a state of equilibrium was observed.

The analysis of the results presented in Table 5 clearly indicates that the experimental data obtained during the adsorption tests were better described by the kinetic equation of the pseudo-second order. This applied in particular to the removal of methylene blue from an alcoholic solution, where the determination coefficients were close to 0.999, higher than the R^2^ values obtained for the pseudo-first-order kinetics. The PSO kinetics was also indicated by the fact that the values of q_cal_ were comparable to those determined experimentally (q_exp_). Based on this, it can be assumed that methylene adsorption involves mainly chemical interactions with the surface of the activated biocarbons derived from walnut shells [52,53].

### 3.5. Adsorption and Electrokinetic Properties of Activated Biocarbons in the Polymers and Surfactant Presence

**Figure 8:**
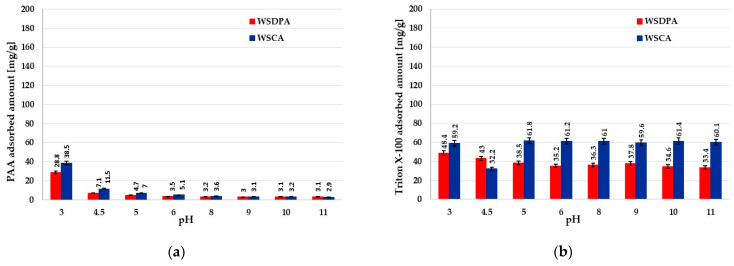

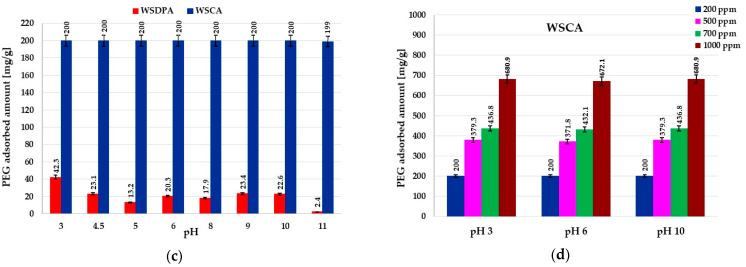
Adsorbed amounts of (**a**) PAA, (**b**) Triton ^TM^ X-100, (**c**) PEG at initial concentration of organic substance 200 ppm and (**d**) PEG at initial concentrations of the polymer 200–1000 ppm on the activated biocarbons’ surfaces as a function of solution pH.

The adsorption abilities of the tested activated biocarbons towards poly(acrylic acid), poly(ethylene glycol) and Triton ^TM^ X-100 within the wide pH range are presented in Figure 8. The analysis of the obtained results indicated that in the case of all adsorbates, the chemically activated WSCA sample was a more effective adsorbent, which was particularly evident for the PEG polymer. The main reasons for such behavior are more favorable textural parameters of this material, i.e., a highly developed specific surface area (about 1360 m^2^/g) and a larger pore size (approx. 3.7 nm) (Table 2). This last parameter is of great importance for the adsorption of macromolecular substances, as it determines the possibility of their penetration into the solid pores, which increases the adsorption capacity [54].

Moreover, the presence of acidic groups on the surface of WSCA activated biocarbon (they were practically absent in the case of the WSDPA sample, Table 2) had a beneficial effect on the adsorption process, as they could participate in the formation of chemical bonds, including hydrogen bonds, with the functional groups of adsorbates. Among the tested systems, this process occurred with the greatest efficiency for nonionic poly(ethylene glycol), and the obtained adsorbed amount reached the maximum value of 680.9 mg/g (Figure 8d). Such a high adsorbed amount could suggest the dense packing of the PEG adsorption layer on the surface of the chemically activated material and its simultaneous expansion towards the bulk solution (the end hydroxyl groups of PEG chains are bound to the surface of the solid via hydrogen bonds). The adsorption affinities of anionic poly(acrylic acid) and nonionic Triton ^TM^ X-100 were considerably lower, and the maximal adsorbed amount obtained for PAA was 38.5 mg/g (Figure 8a), whereas for the surfactant, it was 61.8 mg/g (Figure 8b).

Moreover, polymer samples were characterized by a certain spread (range) of molecular weights, depending, of course, on the polydispersity index of the polymer. Therefore, the average weight was determined; in the case of PAA and PEG, the average molecular weight was equal to 2 kDa. This polydispersity of the polymer caused, in the range of its low concentrations in the solution, all macromolecules to be adsorbed on the solid surface, regardless of their molecular weight. As the polymer concentration increased, macromolecules with lower mass were desorbed by those with higher mass, which, due to their higher adsorption energy, had a greater affinity for the surface. Therefore, a certain type of fractionation occurred in the case of the polydisperse polymer sample, which caused the adsorbed amount to be dependent on the concentration, up to the complete saturation of the surface with polymeric chains of the highest molecular weight available in a given sample.

Analyzing the influence of the solution pH, it can be concluded that the adsorption of pol(ethylene glycol) and Triton ^TM^ X-100 possessing a nonionic nature proceeded independently from this parameter. In such a case, the sign of the solid surface charge (electrostatic forces) did not affect the adsorbed amounts, and it depended on specific interactions between the activated biocarbon surface and the nonionic adsorbate. Only in the case of poly(acrylic acid) was the solution pH effect more visible, especially at pH 3, at which PAA chains assumed the most coiled conformation resulting from the minimal dissociation of its carboxyl groups. Additionally, the greatest adsorption level under acidic pH conditions (in the range 3–4.5), to a lesser extent, may be a consequence of a slight electrostatic attraction between the positively charged surface of carbonaceous material and partly ionized polyelectrolyte macromolecules [55].

**Figure 9:**
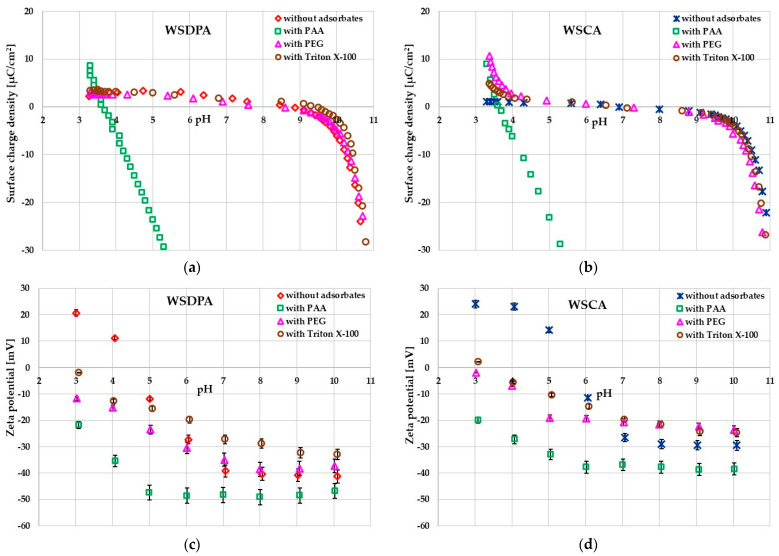
Surface charge density (**a**,**b**) and zeta potential (**c**,**d**) of activated biocarbon particles in their aqueous suspensions containing tested organic compounds.

The specific structure of the adsorption layer formed at the solid/liquid interface determines the values of parameters characterizing EDLs, such as surface charge density and zeta potential. The pH-dependencies of σ_0_ and ζ for the systems without and with polymers and surfactant are presented in Figure 9.

The nonionic adsorbates (poly(ethylene glycol) and Triton ^TM^ X-100) minimally influenced the surface charge density of both activated biocarbons (Figure 9a,b). The lack of electrostatic interactions between their molecules and the adsorbent active sites did not change the number of charged surface groups of carbonaceous materials located in the stiff Stern layer of EDLs (the surface charge density remains in the same level as in the case of the system without adsorbates). Such behavior is typically observed for colloidal suspensions containing nonionic substances [56]. Moreover, the lack of changes in σ_0_ confirms that the dominant interactions in such colloidal systems are hydrogen bridge interactions [57]. A completely different situation occurred after the ionic PAA addition to the dispersed system of activated biocarbons. A considerable decrease in the surface charge density was observed in the poly(acrylic acid) presence. The main reason for this was the accumulation of negatively charged carboxyl groups of the polymer in the by-surface layer of the solution (located in the loop and tail structures of adsorbed chains). Their number was much larger than the dissociated polymer functional groups directly adsorbed in the train structures (which causes the creation of an additional number of positively charged adsorption sites) and therefore, the total effect of the reduction in the surface charge density was observed [58].

In turn, the impact of nonionic adsorbates on the zeta potential of the activated biocarbon particles was more noticeable compared to σ_0_ parameter changes (Figure 9c,d). It was primarily associated with the displacement of the slipping plane from the solid surface caused by the presence of thick adsorption layers of polymer or surfactant molecules with considerable sizes [59]. The slipping plane separates the stationary part of the solution, rigidly bound to the surface, from the diffusive moving part located at a certain distance from the surface of colloidal particles. Such effect also took place in the case of poly(acrylic acid) adsorption. Nevertheless, for PAA, the important role in the zeta potential lowering compared to the system without the polymer was played by its dissociated carboxyl groups, which are located in the slipping plane area together with segments occurring in the loop and tail structures of adsorbed macromolecules [60].

Based on the analysis of the data collected in Table 6, it was found that the obtained activated biocarbons performed quite well in terms of organic dyes, polymers and surfactants removal from aqueous solutions compared to various adsorbents described in the literature. The chemical activation product (WSCA sample) showed much better capacitive characteristics; therefore, further research should focus on optimizing this variant of walnut shells’ activation procedure.

## 4. Conclusions

The conducted studies showed that common walnut shells could be successfully used for the production of inexpensive activated biocarbons, characterized by good sorption abilities towards different types of organic pollutants. The physicochemical properties of carbonaceous materials obtained from this type of biomass were determined by the method of the thermochemical treatment used. Chemical activation of the precursor with H_3_PO_4_ allowed the production of adsorbent with a well-developed surface area (over 1360 m^2^/g), a micro/mesoporous character of the structure and an acidic nature of the surface. In turn, the direct activation of walnut shells with carbon dioxide led to the formation of a microporous material with an alkaline surface, but its textural parameters were much less favorable. The chemically activated sample turned out to be a more effective adsorbent in relation to all tested pollutants. Its sorption capacity towards methylene blue poly(acrylic acid), poly(ethylene glycol) and Triton ^TM^ X-100 reached the levels of 247.1 mg/g, 680 mg/g, 38.5 mg/g and 61.8 mg/g, respectively. Due to the fact that chemically activated carbon had the ability to remove methylene blue from aqueous and alcoholic solutions, it can be potentially used not only in wastewater treatment but also in the decolorization of food industry products. However, additional studies in this area are required (especially in terms of the safety of their use in the food industry).

## Figures and Tables

**Table 1:** Proximate and elemental analysis of the precursor and activated biocarbons [wt.%].

Sample	Moisture	Volatile Matter	Ash	C^daf 1^	H^daf^	N^daf^	S^daf^	O^diff 2^
WS	0.7	80.9	0.9	48.6	5.1	0.3	0.5	45.5
WSDPA	0.0	N/A	3.2	94.2	0.5	0.3	0.0	5.0
WSCA	0.0	N/A	1.8	89.9	2.2	0.1	0.0	7.8

^1^ Dry-ash-free basis; ^2^ calculated by difference; method error ≤ 0.3%.

**Table 2:** Textural and surface properties of activated biocarbons.

Sample	S_t_ [m^2^/g]	S_micr_ [m^2^/g]	V_t_ [cm^3^/g]	V_micr_ [cm^3^/g]	V_micr_/V_t_ [%]	D [nm]	Acidic Groups [mmol/g]	Basic Groups [mmol/g]
WSDPA	401	361	0.27	0.20	74	2.67	0.007	0.949
WSCA	1361	993	1.25	0.56	45	3.68	0.705	0.099

S_t_—total surface area, S_micr_—micropore area, V_t_—total pore volume, V_micr_—micropore volume, V_micr_/V_t_ micropore contribution, method error: 2–5%.

**Table 3:** Comparison of the preparation procedure and textural parameters of activated carbons obtained as a result of chemical and physical activation of walnut shells.

Activation Procedure	Surface Area [m^2^/g]	Total Pore Volume [cm^3^/g]	Reference
H_3_PO_4_ (wt. ratio 2:1), 500 °C, 30 min, grain size 2–4 mm	1361	1.25	Present study
CO_2_, 850 °C, 30 min, grain size 2–4 mm	401	0.27	Present study
KOH (wt. ratio 4:1), 500–800 °C, 30 min, grain size 1.5–2.5 mm	490–2305	0.25–1.13	[41]
CO_2_, 500–800 °C, 60 min, grain size 1.5–2.5 mm	165–697	0.08–0.37	[41]
H_2_O, 700–900 °C, 60–120 min, grain size 1–2 mm	542–1361	0.34–0.74	[42]
CO_2_, 850 °C, 60–480 min, grain size 1–2 mm	542–1304	0.35–0.93	[42]
KOH (wt. ratio ~2.5:1), 800 °C, 180 min, grain size—finely powdered	860	0.45	[43]
H_3_PO_4_ (wt. ratio 7:1), 450 °C, 180 min, grain size 0.5–0.8 mm	1388	1.64	[44]
KOH (wt. ratio 2:1), 800 °C, 60 min, grain size 0.63–1.25 mm	2000	0.82	[45]
CO_2_, 500 °C, 360 min, grain size 4 mm	905	0.34	[46]
ZnCl_2_ (wt. ratio 1:1), 750 °C, 120 min, grain size 0.5–1.0 mm	1360	0.68	[47]

**Table 4:** Langmuir/Freundlich parameters of the equilibrium isotherms of methylene blue adsorption on the activated biocarbons derived from walnut shells.

Sample	q_exp_	Langmuir Model			Freundlich Model		
		q_max_	K_L_	R^2^	K_F_	1/n	R^2^
Aqueous solution							
WSDPA	68.5 ± 2.7	68.9	5.41	0.999	56.9	0.067	0.989
WSCA	247.1 ± 6.1	248.4	1.60	0.997	182.9	0.095	0.979
Ethanolic solution							
WSDPA	21.9 ± 0.8	22.5	152.30	0.992	19.3	0.050	0.476
WSCA	29.7 ± 1.4	28.2	2.19	0.995	17.7	0.194	0.832

q_exp_—the experimental adsorption capacity [mg/g], q_max_—the maximum capacity of the adsorption monolayer [mg/g], K_L_—the Langmuir adsorption equilibrium constant [dm^3^/mg], K_F_—the Freundlich equilibrium constant [mg/g (mg/dm^3^)^1/n^], 1/n—the intensity of adsorption, R^2^—the determination coefficient.

**Table 5:** Kinetic parameters for methylene blue adsorption on the activated biocarbons derived from walnut shells.

Sample	q_exp_	Model PFO				Model PSO			
		q_cal_	k_1_	R^2^	MPSD	q_cal_	k_2_	R^2^	MPSD
**Linear fitting**									
Aqueous solution									
WSDPA	65.2 ± 3.3	15.6	0.0076	0.972	153.1655	65.8	0.0013	0.998	41832.5702
WSCA	214.1 ± 6.4	54.7	0.0187	0.881	1931.868	217.4	0.0009	0.999	537239.1705
Ethanolic solution									
WSDPA	14.3 ± 0.7	3.9	0.0046	0.480	0.6886	13.7	0.0045	0.977	1534.3967
WSCA	24.9 ± 1.2	8.4	0.0088	0.752	88.2837	25.1	0.0027	0.979	5344.2634
**Non-linear fitting**									
Aqueous solution									
WSDPA	65.2 ± 3.3	57.9	0.1293	0.969	0.0240	60.7	0.0044	0.992	0.0085
WSCA	214.1 ± 6.4	204.1	0.1910	0.988	0.0082	209.2	0.0029	0.996	0.0048
Ethanolic solution									
WSDPA	14.3 ± 0.7	11.3	0.2668	0.819	0.1364	12.0	0.0310	0.880	0.1462
WSCA	24.9 ± 1.2	22.2	0.0532	0.954	0.0436	24.9	0.0029	0.985	0.0191

q_exp_—experimental adsorption capacity [mg/g], q_cal_—calculated adsorption capacity [mg/g], k_1_—the pseudo-first-order adsorption rate constant [1/min], k_2_—the pseudo-second-order adsorption rate constant [g/(mg∙min)], R^2^—the determination coefficient, MPSD—Marquardt’s percent standard deviation.

**Table 6:** Adsorption capacity towards methylene blue, poly(acrylic acid), pol(ethylene glycol) and Triton ^TM^ X-100 for various carbonaceous adsorbents.

Adsorbent	Adsorbed Amount [mg/g]	Reference
Methylene blue		
WSDPA	68	This study
WSCA	247	This study
Activated carbon derived from coconut leaves (chemically activated by H_2_SO_4_)	127	[61]
Mesoporous high-surface-area activated carbon from rubber seed pericarp biomass wastes (microwave-assisted H_3_PO4 activation)	348	[62]
Mesoporous activated carbon from agricultural wastes including oil palm frond and palm kernel shell (microwave radiation-assisted K_2_CO_3_ activation)	332	[63]
Zeolite-activated carbon composite from oil palm ash (chemical activation with NaOH and hydrothermal treatment)	144	[64]
Porous graphene–carbon nanotubes composite (hydrothermal reaction)	232	[65]
Sheep and goat dung-derived activated carbon (chemical activation by KOH)	25	[66]
Poly(acrylic acid)		
WSDPA	29	This study
WSCA	38	This study
Peat-based activated carbon (chemical activation with K_2_CO_3_)	265	[33]
Activated carbon obtained from horsetail herb (physical activation with steam and HCl washing)	196	[39]
Nettle herb-based activated carbon (chemical activation with H_3_PO_4_)	273	[54]
Hybrid carbon–mineral nanocomposites with metallic Mn elements	35	[67]
Poly(ethylene glycol)		
WSDPA	43	This study
WSCA	680	This study
Commercial granular activated carbon 207-EA	350	[68]
Commercial activated carbon, Calgon Filtrasob 400	196	[69]
Commercial activated carbon	189	[70]
Commercial activated carbon F-400	480	[71]
Triton X-100		
WSDPA	48	This study
WSCA	62	This study
Activated carbon prepared from waste tires (chemical activation with KOH)	220	[72]
Commercial activated charcoal	230	[73]
Activated carbon obtained from horsetail herb (physical activation with steam and HCl washing)	20	[39]
Carbide-derived carbon	578	[74]

## Data Availability

Data are contained within the article.
